# Supplementary material for: Denoise Pretraining on Nonequilibrium Molecules for Accurate and Transferable Neural Potentials
Source: J Chem Theory Comput. 2023 Jun 30;19(15):5077–87. doi: 10.1021/acs.jctc.3c00289 (PMC10413865; doi:10.1021/acs.jctc.3c00289)
Supplement: Supplementary file 1 — ct3c00289_si_001.pdf [file ct3c00289_si_001.pdf]

Supporting Information

Denoise Pretraining on Nonequilibrium Molecules for Accurate and Transferable Neural Potentials

Yuyang Wang,[†,‡] Changwen Xu,[†] Zijie Li,[†] and Amir Barati Farimani[*,†,‡,¶,§]

†*Department of Mechanical Engineering, Carnegie Mellon University, Pittsburgh, PA 15213, USA*

‡*Machine Learning Department, Carnegie Mellon University, Pittsburgh, PA 15213, USA*

¶*Department of Materials Science and Engineering, Carnegie Mellon University, Pittsburgh, PA 15213, USA*

§*Department of Chemical Engineering, Carnegie Mellon University, Pittsburgh, PA 15213, USA*

E-mail: barati@cmu.edu

S1 Details of MD22

The MD22[1] dataset is composed of AIMD trajectories covering seven systems of biomolecules (proteins, carbohydrates, and nucleic acids) or supramolecules (buckyball catcher and nanotube). Table S1 lists the molecule, data size, and number of atoms per molecule of each system. The diverse molecular size and flexibility of the dataset provide challenges for the exploration of neural potentials, making it a suitable benchmark to advance research on ML-based force fields.

Table S1: Details of MD22[1] dataset.

Molecule	# Data	# Atoms
Ac-Ala3-NHMe	85,109	42
Docosahexaenoic acid	69,753	56
Stachyose	27,272	87
DNA base pair (AT-AT)	20,001	60
DNA base pair (AT-AT-CG-CG)	10,153	118
Buckyball catcher	6,102	148
Double-walled nanotube	5,032	370

S2 Fine-tuning Details

For ANI-1[2] and ANI-1x,[3] all models are fine-tuned for 10 epochs with batch size 256 and maximal learning rate 2×10^{-4}. For SPICE,[4] all models are fine-tuned with the same batch size and learning rate as ANI-1 and ANI-1x but are trained for 50 epochs. For ISO17,[5] all models are fine-tuned for 50 epochs with batch size 64 and maximal learning rate 2×10^{-4}. The hyperparameters used for fine-tuning GNNs on MD22 dataset[1] are listed in Table S2, which are determined according to the model performance and computation cost and efficiency.

Table S2: Fine-tuning details of GNNs on MD22[1] dataset.

Model	Molecule	Epoch	Batch size	Max LR
SchNet	Ac-Ala3-NHMe	100	64	2×10^{-4}
	Docosahexaenoic acid	100	64	2×10^{-4}
	Stachyose	100	64	2×10^{-4}
	DNA base pair (AT-AT)	100	64	2×10^{-4}
	DNA base pair (AT-AT-CG-CG)	100	64	2×10^{-4}
	Buckyball catcher	100	64	2×10^{-4}
	Double-walled nanotube	100	16	5×10^{-5}
SE(3)-Transformer	Ac-Ala3-NHMe	50	64	2×10^{-4}
	Docosahexaenoic acid	50	64	2×10^{-4}
	Stachyose	50	64	2×10^{-4}
	DNA base pair (AT-AT)	50	64	2×10^{-4}
	DNA base pair (AT-AT-CG-CG)	50	64	2×10^{-4}
	Buckyball catcher	50	32	2×10^{-4}
	Double-walled nanotube	50	16	5×10^{-5}
EGNN	Ac-Ala3-NHMe	100	32	2×10^{-4}
	Docosahexaenoic acid	100	32	2×10^{-4}
	Stachyose	100	32	2×10^{-4}
	DNA base pair (AT-AT)	100	32	2×10^{-4}
	DNA base pair (AT-AT-CG-CG)	100	32	2×10^{-4}
	Buckyball catcher	100	32	2×10^{-4}
	Double-walled nanotube	100	16	5×10^{-5}
TorchMD-Net	Ac-Ala3-NHMe	100	32	2×10^{-4}
	Docosahexaenoic acid	100	32	2×10^{-4}
	Stachyose	100	32	2×10^{-4}
	DNA base pair (AT-AT)	100	32	2×10^{-4}
	DNA base pair (AT-AT-CG-CG)	100	32	2×10^{-4}
	Buckyball catcher	100	32	2×10^{-4}
	Double-walled nanotube	100	16	5×10^{-5}

S3 Implementation of GNNs

SchNet[6] is implemented based on the PyG [a] and MDSim [b]. SE(3)-Transformer[7] is implemented based on the public code provided by the authors [c]. EGNN[8] is also implemented based on the original public code [d]. Similarly, we adapt the public code of TorchMD-Net[9] from the original work [e]. We initialize the hyperparameters based on the default settings reported in the original works and customize the settings to fit the models into the GPU. Table S3 lists the number of parameters as well as the symmetry principle for each model implemented in this work. Also, Table S4, S5, S6, and S7 show the detailed hyperparameters of SchNet, SE(3)-Transformer, EGNN, and TorchMD-Net, respectively.

Table S3: Number of parameters for each GNN implemented in this work.

Model	Symmetry Principle	# Parameters
SchNet	E(3)-invariant	1.42M
SE(3)-Transformer	SE(3)-equivariant	0.24M
EGNN	E(3)-equivariant	2.38M
TorchMD-Net	E(3)-equivariant	5.11M

Table S4: Hyperparameter of SchNet.

Hyperparameter	Value
# interaction layers	5
# hidden channels	256
# filters	256
# Gaussian function	32

[a] https://github.com/pyg-team/pytorch_geometric/blob/master/torch_geometric/nn/models/schnet.py

[b] https://github.com/kyonofx/MDsim

[c] https://github.com/FabianFuchsML/se3-transformer-public

[d] https://github.com/vgsatorras/egnn

[e] https://github.com/torchmd/torchmd-net

Table S5: Hyperparameter of SE(3)-Transformer.

Hyperparameter	Value
# layers	3
# channels	8
# degrees	4
# attention heads	2

Table S6: Hyperparameter of EGNN.

Hyperparameter	Value
# layers	5
# hidden channels	256
Residual connection	`True`
Attention	`True`
Normalizing message	`True`

Table S7: Hyperparameter of TorchMD-Net.

Hyperparameter	Value
# layers	6
# hidden channels	256
# attention heads	8
# RBF functions	32
Activation function	`SiLU`

S4 Computational Efficiency of Different GNNs

The computational efficiencies of different GNN models included in this work are shown in Table S8. All results are benchmarked on one NVIDIA RTX A6000 GPU with Intel Core i9-10900X CPUs.

Table S8: Time efficiency of different equivariant GNN models. (a) Pretraining time (in hours) per epoch on the combination of ANI-1 and ANI-1x, (b) fine-tuning time (in hours) per epoch on ANI-1x, and (c) total inference time (in minutes) on the test set of ANI-1x dataset.

Model	Pretrain (h)	Fine-tune (h)	Inference (min)
SchNet	1.09	0.24	1.11
SE(3)-Transformer	76.38	5.43	23.82
EGNN	1.64	0.43	1.71
TorchMD-Net	4.08	0.59	4.55

S5 Investigation of Fine-tuning Epochs

Table S9 show the performance of EGNN and TorchMD-Net on ANI-1x when fine-tuned for different epochs. Both GNNs that are pretrained or not are reported. It is observed that fine-tuning for more epochs can improve the prediction accuracy. However, even trained for more epochs, GNNs that are not pretrained cannot compete with pretrained ones. This further demonstrates the effectiveness of denoise pretraining for molecular potential predictions.

Table S9: Performance of different GNNs on ANI-1x with different fine-tuning epochs.

Model	Pretrain	Epochs	RMSE (kcal/mol)	MAE (kcal/mol)
EGNN		10	7.35	5.30
EGNN		15	6.30	4.55
EGNN	✓	10	4.94	3.49
EGNN	✓	15	4.70	3.32
TorchMD-Net		10	2.27	1.50
TorchMD-Net		15	1.79	1.17
TorchMD-Net	✓	10	1.54	1.01
TorchMD-Net	✓	15	1.43	0.93

S6 Uncertainty in Fine-tuning

In this section, we report uncertainty in the performance metrics of neural potential predictions with different random seeds for data splitting. We randomly split each molecular data in MD22 with three different random seeds and report the mean and standard deviation over the three runs. Table S10 and S11 list RMSE and MAE of EGNN and TorchMD-Net. It is observed that the pretrained GNNs consistently perform better than the non-pretrained counterparts.

Table S10: Mean and standard deviation of RMSE (kcal/mol) over three runs with different data splitting on MD22.

Task	$\text{EGNN}_{\text{scratch}}$	$\text{EGNN}_{\text{pretrain}}$	$\text{TorchMD-Net}_{\text{scratch}}$	$\text{TorchMD-Net}_{\text{pretrain}}$
Ac-Ala3-NHMe	0.180(0.011)	0.114(0.002)	3.136(0.159)	0.541(0.036)
DHA	0.308(0.025)	0.151(0.002)	4.798(0.045)	1.254(0.107)
Stachyose	0.819(0.078)	0.633(0.035)	7.177(0.354)	3.548(0.177)
AT-AT	0.564(0.048)	0.232(0.011)	4.524(0.010)	1.590(0.961)
AT-AT-CG-CG	0.748(0.040)	1.083(0.052)	11.390(0.368)	5.134(0.462)
Buckyball catcher	1.292(0.051)	1.668(0.059)	13.965(0.277)	5.721(1.801)
Nanotube	26.112(2.372)	9.812(0.065)	62.228(2.223)	21.849(4.794)

Table S11: Mean and standard deviation of MAE (kcal/mol) over three runs with different data splitting on MD22.

Task	$\text{EGNN}_{\text{scratch}}$	$\text{EGNN}_{\text{pretrain}}$	$\text{TorchMD-Net}_{\text{scratch}}$	$\text{TorchMD-Net}_{\text{pretrain}}$
Ac-Ala3-NHMe	0.125(0.008)	0.079(0.001)	2.376(0.145)	0.372(0.022)
DHA	0.206(0.013)	0.105(0.001)	3.709(0.028)	0.878(0.065)
Stachyose	0.572(0.033)	0.438(0.015)	5.643(0.271)	2.663(0.176)
AT-AT	0.397(0.035)	0.164(0.003)	3.528(0.012)	1.145(0.729)
AT-AT-CG-CG	0.785(0.025)	0.532(0.025)	9.022(0.302)	4.025(0.353)
Buckyball catcher	1.168(0.134)	0.907(0.067)	12.062(1.903)	4.252(1.218)
Nanotube	20.118(1.141)	7.798(0.139)	46.790(0.566)	17.091(3.539)

References

(1) Chmiela, S.; Vassilev-Galindo, V.; Unke, O. T.; Kabylda, A.; Sauceda, H. E.; Tkatchenko, A.; Müller, K.-R. Accurate global machine learning force fields for molecules with hundreds of atoms. *Science Advances* **2023**, *9*, eadf0873.

(2) Smith, J. S.; Isayev, O.; Roitberg, A. E. ANI-1: an extensible neural network potential with DFT accuracy at force field computational cost. *Chemical science* **2017**, *8*, 3192–3203.

(3) Smith, J. S.; Zubatyuk, R.; Nebgen, B.; Lubbers, N.; Barros, K.; Roitberg, A. E.; Isayev, O.; Tretiak, S. The ANI-1ccx and ANI-1x data sets, coupled-cluster and density functional theory properties for molecules. *Scientific data* **2020**, *7*, 1–10.

(4) Eastman, P.; Behara, P. K.; Dotson, D. L.; Galvelis, R.; Herr, J. E.; Horton, J. T.; Mao, Y.; Chodera, J. D.; Pritchard, B. P.; Wang, Y., et al. SPICE, A Dataset of Drug-like Molecules and Peptides for Training Machine Learning Potentials. *Scientific Data* **2023**, *10*, 1–11.

(5) Schütt, K.; Kindermans, P.-J.; Sauceda Felix, H. E.; Chmiela, S.; Tkatchenko, A.; Müller, K.-R. Schnet: A continuous-filter convolutional neural network for modeling quantum interactions. *Advances in neural information processing systems* **2017**, *30*.

(6) Schütt, K. T.; Sauceda, H. E.; Kindermans, P.-J.; Tkatchenko, A.; Müller, K.-R. Schnet–a deep learning architecture for molecules and materials. *The Journal of Chemical Physics* **2018**, *148*, 241722.

(7) Fuchs, F.; Worrall, D.; Fischer, V.; Welling, M. Se (3)-transformers: 3d roto-translation equivariant attention networks. *Advances in Neural Information Processing Systems* **2020**, *33*, 1970–1981.

(8) Satorras, V. G.; Hoogeboom, E.; Welling, M. E (n) equivariant graph neural networks. International conference on machine learning. 2021; pp 9323–9332.

(9) Thölke, P.; De Fabritiis, G. TorchMD-NET: Equivariant Transformers for Neural Network based Molecular Potentials. *arXiv preprint arXiv:2202.02541* **2022**,
